# Learning from Nature: Bioinspired Chlorin-Based Photosensitizers Immobilized on Carbon Materials for Combined Photodynamic and Photothermal Therapy

**DOI:** 10.3390/biomimetics5040053

**Published:** 2020-10-14

**Authors:** Lucas D. Dias, Ivan S. Mfouo-Tynga

**Affiliations:** São Carlos Institute of Physics, University of São Paulo, São Carlos 13566-590, Brazil; tivansdavids2012@gmail.com

**Keywords:** bioinspired photosensitizer, cancer, carbon nanomaterial, chlorin, photodynamic therapy, photothermal therapy

## Abstract

Chlorophylls, which are chlorin-type photosensitizers, are known as the key building blocks of nature and are fundamental for solar energy metabolism during the photosynthesis process. In this regard, the utilization of bioinspired chlorin analogs as photosensitizers for photodynamic therapy constitutes an evolutionary topic of research. Moreover, carbon nanomaterials have been widely applied in photodynamic therapy protocols due to their optical characteristics, good biocompatibility, and tunable systematic toxicity. Herein, we review the literature related to the applications of chlorin-based photosensitizers that were functionalized onto carbon nanomaterials for photodynamic and photothermal therapies against cancer. Rather than a comprehensive review, we intended to highlight the most important and illustrative examples over the last 10 years.

## 1. Introduction

Photodynamic therapy (PDT) is a light-based therapy that uses photosensitizing molecules (PSs), light of an appropriate wavelength, and molecular oxygen (O_2_) to impart cytotoxicity via oxidative reactions, which are able to combat cancer cells (solid tumor) [1,2] and infectious diseases [3,4,5]. Regarding its mechanism, a light-activated PS transfers its excited-state energy to the O_2_ for generating reactive oxygen species (ROS) and singlet oxygen (^1^O_2_), which are able to destroy cancerous cells via oxidative pathways [6]. Among the PSs used so far, we highlighted the bioinspired tetrapyrrole macrocycle photosensitizers, such as porphyrin, chlorin, and bacteriochlorin derivatives [7]. These types of photosensitizers were bioinspired by vital natural molecules, for instance, heme, chlorophyll *a*, and bacteriochlorophyll *a*, and have been reported as highly active photosensitizers [8].

**Figure 1:**
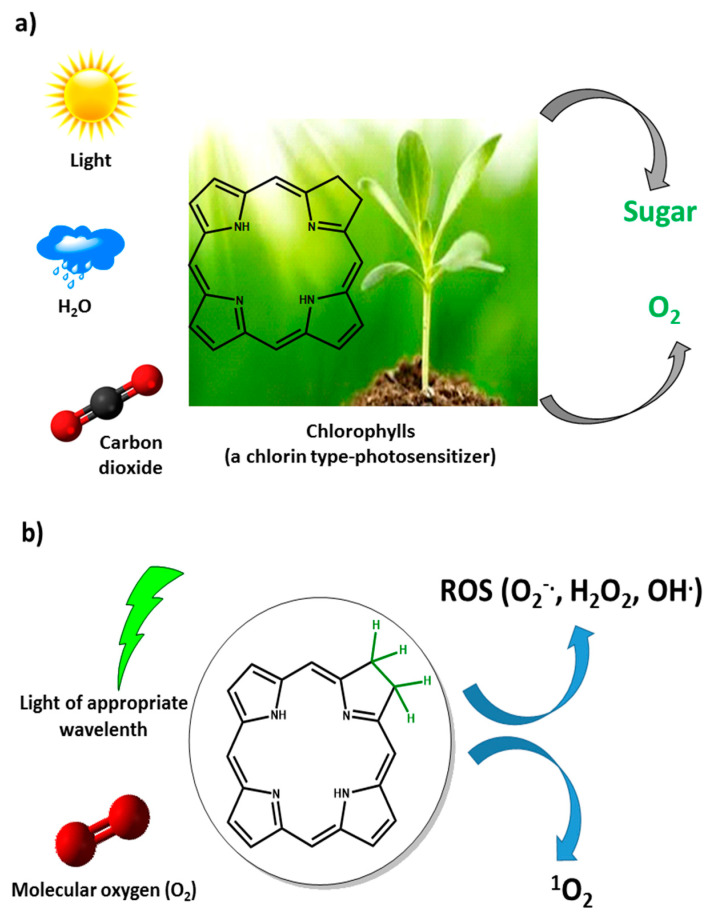
(**a**) General photosynthesis process; (**b**) Representation of a simple photodynamic therapy protocol. ROS: reactive oxygen species.

In nature, the interaction of light (solar energy) with a photosensitizing molecule (chlorophyll *a*) results in the production of sugar and O_2_ via a photosynthesis process [9]. Similarly, in photodynamic therapy, the interaction of light (energy) with a photosensitizing molecule results in the production of other molecules, in this case, ROS. This is a classic example where people have been using bioinspiration in nature to generate benefits for society (Figure 1).

Moreover, in recent years, the combination of photodynamic and photothermal therapies (PTTs) by using photosensitizers and carbon nanomaterials combined (carbon nanotube, graphene, fullerene, carbon dot) have been widely studied/tested, showing excellent results [10,11,12,13]. These carbon-based nanomaterials are considered promising photosensitizers due to their optical properties, high biocompatibility, and low toxicity [14,15]. This strategy, which involves the combination of a carbon nanomaterial with other organic photosensitizers, utilizes covalent (more stable and using specific linkage) or non-covalent (electrostatic forces, e.g., π–π stacking, van der Waals forces, hydrogen bonding, or hydrophobic interaction) linkages [16,17,18,19,20].

Herein, we review the literature related to the applications of bioinspired chlorin-based photosensitizers that were immobilized onto carbon nanomaterials for photodynamic therapy against cancer. Rather than a comprehensive review, we intended to highlight the most important and illustrative examples from the last 10 years.

## 2. Photodynamic and Photothermal Therapy: Mechanism and Applications

### 2.1. Mechanisms of Photodynamic and Photothermal Therapy

**Figure 2:**
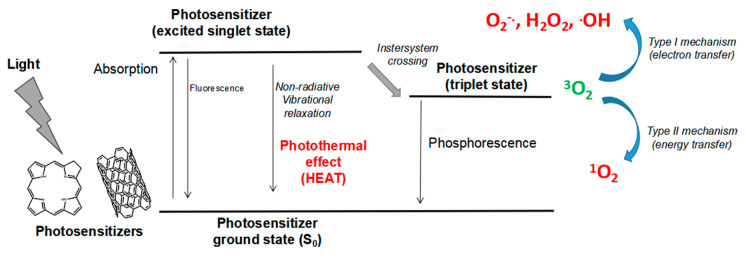
Simplified Jablonski diagram depicting the different energy levels induced after light irradiation. The phototherapeutic agent (photosensitizer) is excited from ground level (S_0_) to the singlet excited state, where it may lose its absorbed energy through fluorescence or non-radiative reactions. Effective agents undergo intersystem crossing and conversion into the triplet excited state, which enables photoreactions to take place [24].

After PS photoactivation, PDT induces tumor and vascular damage, and mainly leaves normal tissues unaffected, due to the improved selectivity, as seen with later generations of PSs. Light of a suitable wavelength and intensity reach the PSs to undergo a series of photochemical reactions [21]. Light energy as a photon is transferred to the inert PS, changing it into activated or excited PS. In this form, the PS can lose the received energy via various possible pathways. The fluorescent phenomenon may occur and lead to the detection and delineation of the tumor target site, but this method is commonly underutilized. An excited PS may lose energy via undergoing type I photochemical reactions, yielding destructive free radicals, or via a type II photochemical process (singlet oxygen) (Figure 2). It is difficult to determine which of these mechanisms (type I or II) is more predominant; both types of reactions can happen depending on three singular features: (i) the presence of oxygen, (ii) photosensitizer concentration, and (iii) PS type [22]. The singlet oxygen species has a half-life span of 40 nanoseconds, which enables it to induce damage within a radius of 20 nanometers, leading to further induction of a cascade of cellular events that cause local, regional, and systemic damage and eradication of tumor [23].

In clinical scenarios, a two-step procedure is performed, starting with systemic administration of PSs via intravenous injection or topical application. The second step is exposing the sensitized tissue to the light to induce a series of photochemical reactions, oxygen reactive species, and subsequent destruction of the tissue under treatment [25]. Antimicrobial photodynamic therapy (aPDT) is highly recommended for cases of increased antimicrobial resistance. There are studies in the literature that reported the inactivation of both Gram-positive and -negative bacteria (up to 7 log units) by using this light-based therapy [26,27]. Activation of PSs can considerably reduce the number of bacteria and represents a promising approach to antimicrobial treatment [26]. The bactericidal effect of aPDT can be further enhanced by applying aPDT after the employment of certain enzymes or plant extracts as a combination therapy against biofilms [26].

PTT is also a minimally invasive and localized modality that utilizes a PS, which is able to absorb and convert energy into heat. This photothermal effect (i.e., heat generation) arises as a result of non-radiative relaxation processes, as presented in Figure 2. The type of absorbing agent (PS), light wavelength, and delivery (interstitial or non-invasive) are all determinants for the energy absorption and subsequent induction of PTT in tumors. Photothermal reactions are enhanced by using photothermal transducers that exhibit strong absorption in NIR and ensure that the generated heat is selectively dissipated in the tumor tissue and not into surrounding tissue [28]. The light device should uniformly deliver heat to the PS that is pre-localized in the targeted tumor, thus increasing the temperature and leaving the surrounding tissues unaffected. Above 41 °C, the photothermal effects become detrimental, where the damaging effects propagate from the core of the tumor and create a thermal gradient at the edges. The mechanisms of PTT for complete or partial thermal energy generation depend on the physical-chemical characteristics of the agent, including photothermal conversion efficiency, low power density, and biocompatibility, as well as photostability and absorption in the NIR region [29]. Photothermal excitation initiates PTT via the direct generation of heat after the irradiation. NIR light offers a better light penetration depth into tumor tissue from about 10 mm to a few centimeters to cause ablation and killing via hyperthermia [30]. The extent of the induced damage depends on the distribution of the agent within the tumor. Certain nanomaterials (e.g., gold nanoparticles, graphene oxide sheets, carbon nanotubes) are becoming famous for their roles as energy absorbers and heat-conversion mediators [28]. Necrosis and apoptosis are often cited as the main or predominantly stated induced damaging mechanisms when using high and low energy irradiation, respectively [31]. The induction of necrosis appears as the main response when using high light doses, while apoptosis seems to be predominant at a relatively low light dose. After PDT and mulling over different response probabilities of death, apoptosis and senescence are considered as the main physiological modes, while necrosis and stress-induced death are the major provoked pathological modes. Other death modes appeared as variant forms of those physiological and pathological responses [32]. The necrotic response seems to always be appearing, especially at the later stages of the flow cytometry analysis using Annexin-V PI staining detection. Optimal conditions need to be carefully determined in order to prevent the development of tumor resistance and recurrence. Finally, effective responses strongly depend on the used model (in vitro or in vivo), type of PS, and treatment protocol.

**Figure 3:**
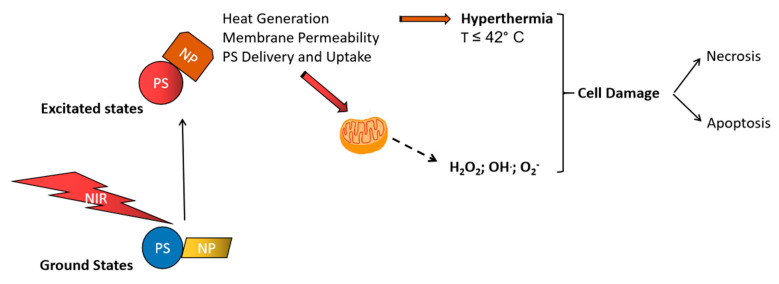
In combination therapy, both photodynamic and photothermal therapy (PTT) and photodynamic therapy (PDT) agents are irradiated and excited to an upper electronic level, where the PTT agent generates heat that facilitates cell membrane permeability and more PDT agent release and uptake into tumor tissue. Hyperthermia and free radical generation cause ablation and tumor damage and killing through necrosis and/or apoptosis. PS: photosensitizing molecule, NP: nanoparticle; NIR: near-infrared region.

When used in combination, PTT resulted in more tumor-killing and enhanced therapeutic outcomes, which were associated with the use of 2D nanomaterials as photothermal agents. Photosensitizers used in PDT are able to generate reactive oxygen species that lead to tumor-killing but still possess limitations, including low selectivity, poor water solubility, and moderate delivery to targeted tumor locations. When combined with certain nanomaterials that act as PTT-mediating agents, enhanced theranostic effects can be achieved, as well as improved targeting and controlled delivery in targeted tumor sites [33]. The heat generated by PTT agents stimulates cell membrane permeability, drug delivery, and the subsequent better uptake of PDT agents by tumors, which are destroyed by PTT-induced hyperthermia and enhanced PDT effects (Figure 3). Combining therapies, such as PTT and PDT, could lead to synergic effects, resulting in effective and multifunctional theranostic applications [34,35].

### 2.2. Anticancer and Antimicrobial Applications of PDT

PDT-mediated tumor damage induces both programmed and non-programmed cell death pathways [36]. Carcinogenesis stimulates genetic mutations and the disturbance of life-sustaining mechanisms. To allow for better selectivity and damage to malignant tissues, certain mechanisms, such as receptor binding, lipid binding, uptake via tyrosine kinase or epidermal growth factor receptor, diffusion, and bio-distribution, are activated to mediate cellular damaging events, which are responsible for the initiation of cancer therapeutic action [37,38,39]. The PS organelle or subcellular localization is the initiation site of PDT-mediated damage and depends upon the kind of PS used. A well-known derivative of porphyrin, Photofrin^®^, is taken up and preferentially accumulated in cell membranes and other organelle membranes, such as mitochondria. Some amphiphilic PSs also accumulate in mitochondria, while others have the Golgi apparatus and endoplasmic reticulum as their preferential localization sites [40,41]. At high light doses, the tumor tissues are rapidly destroyed through a non-programmed pathway following a necrotic manner. During this execution, sub-cellular membranes are destroyed, leading to the excessive release of calcium ions and metabolic byproducts, which causes multiple forms of dysfunction and damage beyond repair [42,43,44]. The release of apoptogenic molecules, cytokines, and ROS cause lethal damage in neighboring tissues, known as the bystander effect, which can propagate to a certain extent [45,46]. PDT may induce programmed cell death through the activation of autophagic cell death and the induction of apoptosis. A low light dose triggers sequential apoptosis events, which start to cease cellular functions and cause cell death in the end. No major bystander or immune effects are required, as no excessive photodamaging effects are usually produced with a low light dose. Apoptosis is a well-conserved mechanism that eliminates damaged cells to preserve the integrity and vitality of tissues. Apoptosis operates well in both tumor and normal cells, as well as in other species like bacteria [47]. However, cancer may promote anti-apoptotic events over proapoptotic ones, hence it is essential to strategically apply target or combined treatments to ensure the facilitation of proapoptosis [48,49]. PDT is lethal and principally affects tumor tissues, leaving surrounding tissues unaffected and the PSs are cleared out and not retained in normal cells.

With an increasing number of pathogens becoming resistant, the common antibiotics are losing their efficacy as the need for an innovative technique to inactivate pathogens without inducing any resistance continues to rise. aPDT is recommended for cases of increased antimicrobial resistance and offers many advantages, including a broader targeting range of actions (bacteria, protozoa, fungi), reduced adverse effects, and antibacterial effects against antibiotic-resistant strains [50]. aPDT is an effective modality for in vitro, in vivo, and clinical applications (e.g., dentistry field) [51,52], and its efficacy has also been proven for localized and superficial infections [53].

## 3. Bioinspired Photosensitizers

**Figure 4:**
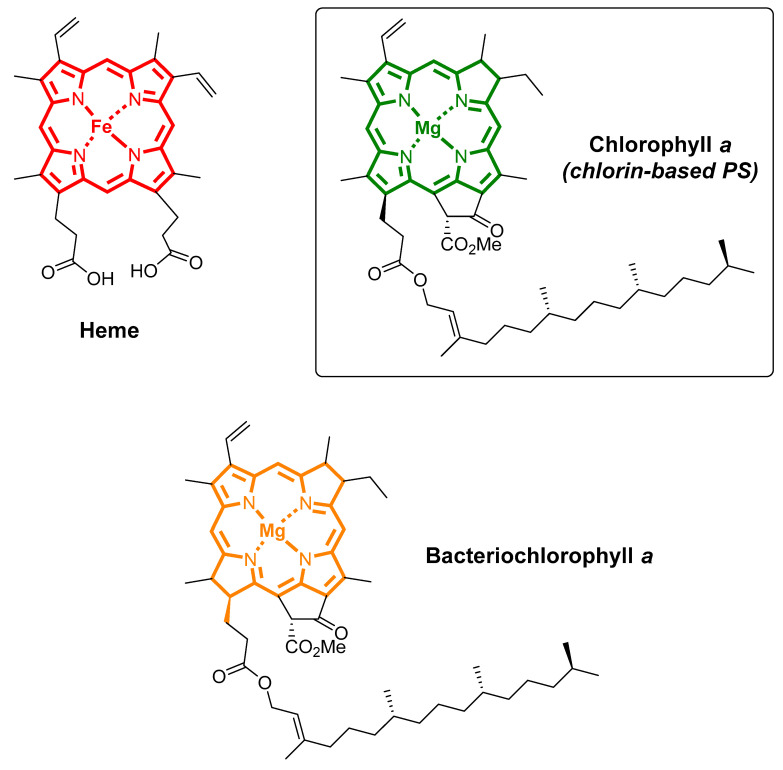
Tetrapyrrolic macrocycles that are involved in natural processes.

Natural molecules, such as chlorophylls, heme, and cobalamin, are considered the “gold standard” that inspires chemists to precisely modulate effective photosensitizers for PDT applications [54,55], photocatalysts/catalysts for chemical processes [56,57], and molecular electronics [58,59]. These tetrapyrrolic macrocycle types are commonly found in nature and provide a key biochemical role in many natural processes. An example of porphyrin in biological processes is the heme present in cytochrome P-450 monooxygenase enzymes [60], hemoglobin, and myoglobin for the transport and storage of O_2_ [61]. Chlorophyll is responsible for the green color of plants, algae, and cyanobacteria, and bacteriochlorophyll *a*, a bacteriochlorin derivative, is involved in photosynthesis in some bacteria. Overall, these tetrapyrrolic macrocycles are considered the “pigments of life” [62] (Figure 4).

**Figure 5:**
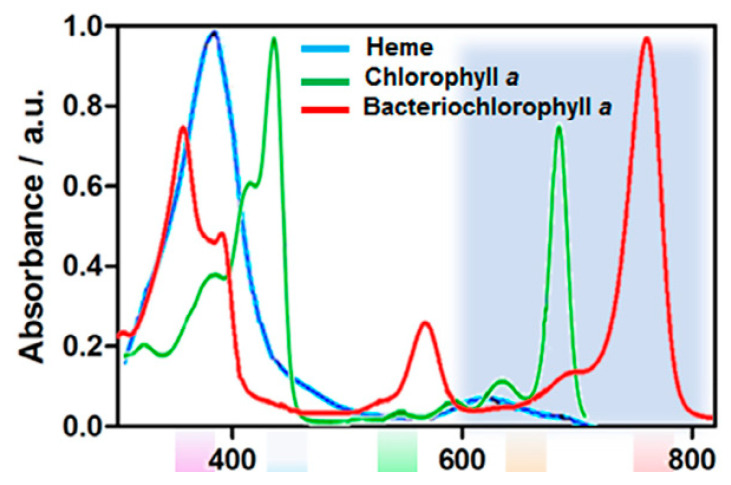
UV-vis absorption spectra of tetrapyrrolic macrocycles that are involved in natural processes. Reprinted from Pucelik et al. [8] with permission (open access) from Elsevier, Copyright 2020.

Regarding the PDT field, tetrapyrrolic macrocycles are one of the most applied photosensitizers, especially the porphyrin and chlorins ones. Their structural and optical characteristics are based on four pyrrole rings and methine bridges, resulting in an aromatic macrocycle [7]. The general UV-vis absorption spectra of porphyrin, chlorin, and bacteriochlorin are presented in Figure 5. The range of spectra between 650 and 850 nm, where light penetrates tissue up to 1–3 cm, has been termed the phototherapeutic window for systemic in vivo applications (e.g., solid tumors) [63], but for antimicrobial photodynamic therapy, absorption of light in the blue region is sufficient [64].

Porphyrin photosensitizers show an absorption band in the region of 400 nm (Soret band) and other small bands in the region of 630 nm [7,8]. Chlorin-type photosensitizers have one of its double bonds in the pyrrole ring reduced, which results in a strong absorption band in the violet-blue region (≈380–450 nm), also known as the B or Soret band, and a moderate band in the red region (≈600–700 nm), known as the Q band [7,8]. While bacteriochlorins have two reduced double bonds in the pyrrole rings, resulting in a strong absorption in the near-infrared region (NIR), this class of photosensitizers shows the tendency toward rapid phototransformation, low stability, and conversion to chlorin and/or porphyrin precursors [65,66]. However, there are some examples in the literature describing highly active bacteriochlorins as photosensitizers in PDT protocols, e.g., redaporfin (LUZ11) [65,66]. One way to overcome this instability is via the presence of electron-withdrawing substituents, the insertion of appropriate metal ions into the macrocycle, and the presence of exocyclic rings in the macrocycle [66].

Among them, this review paper focuses on chlorin photosensitizers. This class of photosensitizers can be obtained via four main routes: (i) the isolation of naturally occurring chlorophylls; (ii) synthesis using porphyrin as precursors (via hydrogenation, annulation, cycloaddition, and breaking and mending); (iii) semi-synthesis of chlorins, beginning with naturally occurring chlorophylls; (iv) de novo synthesis of gem-dialkylchlorins, wherein the reduced ring is linked to the acyclic precursors of the chlorin [67]. As the most studied PS, emphasis will be put on chlorin e6 (Ce6) and its derivatives.

**Figure 6:**
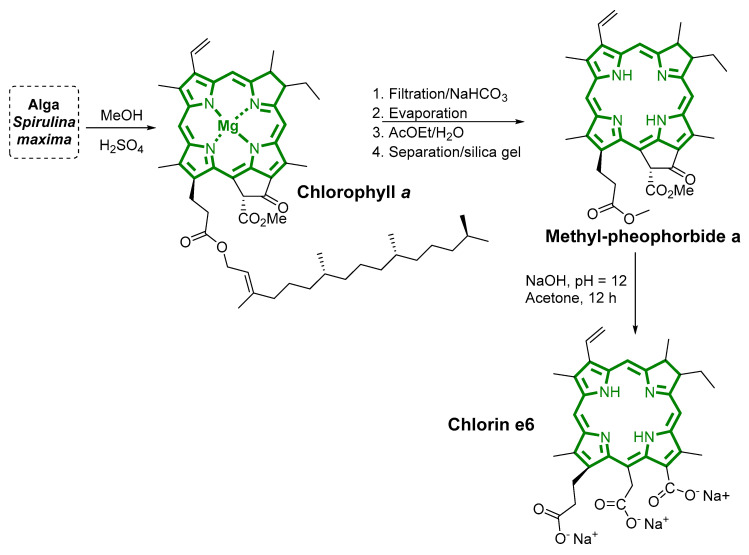
Methodology for the chlorin e6 extraction from *Spirulina maxima*.

As a selected example, Uliana and co-authors described obtaining chlorin e6 from *Spirulina maxima* by using a simple extraction method (Figure 6) [68]. First, a methyl-pheophorbide a derivative was obtained through extraction from dried *Spirulina maxima*, followed by filtration, neutralization using sodium bicarbonate, and purification via silica gel flash chromatography. The methyl-pheophorbide a derivative was obtained in 0.8% yield from natural alga. Then, the chlorin e6 was obtained in 89% yield via the basic hydrolysis of methyl-pheophorbide in the presence of an aqueous NaOH solution and acetone as the solvent.

**Figure 7:**
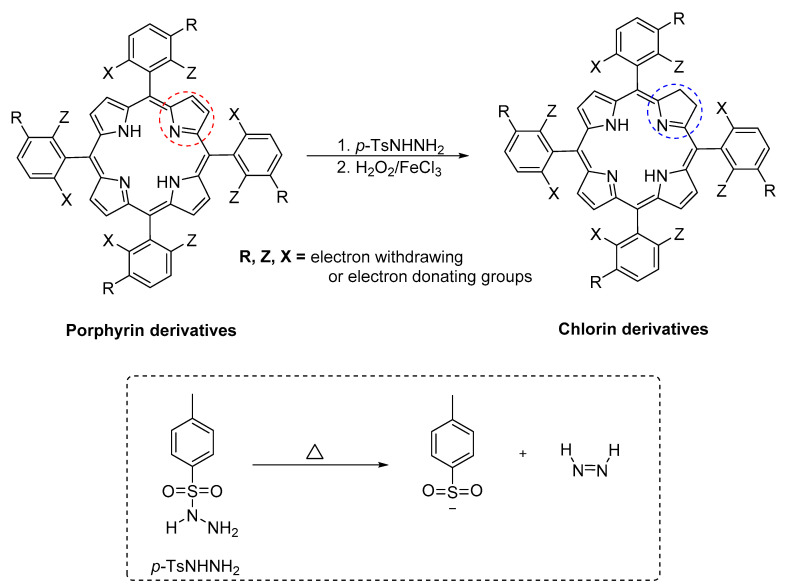
Methodology for the synthesis of chlorin via the hydrogenation of a porphyrin precursor by using *p*-TsNHNH_2_. Red circle: pyrrole ring; Blue circle: pyrrole ring reduced.

Another approach to obtaining chlorin photosensitizers is via synthetic modulation by using porphyrin as a precursor. In 2012, Pereira and co-authors reported a sustainable methodology for chlorin synthesis via the reduction of porphyrin precursors using *p*-toluenesulfonylhydrazide in the total absence of solvents and bases. In this method, the desired porphyrin precursors were mixed with *p*-toluenesulfonylhydrazide and heated up to 140 °C in an evacuated tube (0.1 bar) [69]. When the ratios of (30:1) and (8:1) of TsNHNH_2_:porphyrin were used, the bacteriochlorin and a mixture of porphyrin, chlorin, and bacteriochlorin were obtained. When a ratio of 15:1 was used, a chlorin derivative with 10–20% of bacteriochlorin was obtained. The authors also described that when using a mixture of H_2_O_2_ and FeCl_3_ as the oxidants, selectivity for chlorin was observed (Figure 7).

Overall, so far there are many methodologies for obtaining chlorin photosensitizers, as described by Lindsey in 2017 [67]. The choice of method and target chlorin molecular structure should be based on some factors: (i) the type of PDT application (antimicrobial or anticancer photodynamic protocol); (ii) cost of the process; (iii) appropriate photo, physical, and chemical properties (solubility, stability, log P, the quantum yield of triplet state, etc.).

## 4. Carbon Materials Applied in Photodynamic/Photothermal Therapy

**Figure 8:**
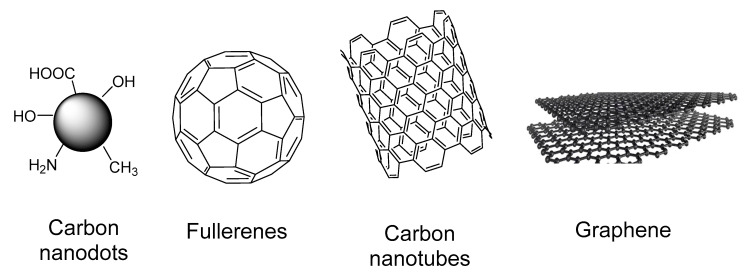
General structures of some carbon nanomaterials.

Carbon is the most versatile element in the periodic table [70] and its ability to hybridize in sp, sp^2^, and sp^3^ configurations opens the way for the existence of a number of allotropes. So far, there are three naturally occurring allotropes of carbon (diamond, amorphous carbon, and graphite), and the synthetic ones include graphene, carbon nanotubes, fullerenes, carbon nanohorns, and nanodiamonds [71] (Figure 8).

The properties of carbon materials (dimensions, hybridization, electrical conductivity, Young’s modulus) make them applicable in many fields ranging from materials science to biomedical applications [72]. Among these advantages, these carbon materials show strong absorption in the visible-NIR regions, which require relatively lower energy and laser intensity for photoinduction and a large surface for the development of new generations of anticancer systems [73].

Upon light activation, these carbon materials are capable of generating ROS through type I and II photodynamic reactions (PDR). They are designated as potential candidates for PDT applications. Both carbon-nanotube- and graphene-mediated PDR generate a significant amount of heat, thus they are considered as PTT agents [74]. Carbon-based nanoparticles are extensively used for PDT applications due to their distinctive optical characteristics, good biocompatibility, and tunable systemic toxicity [11]. The discovery of fullerene corresponds to the beginning of the study in the field that focuses on carbon nanomaterials. Fullerene has a soccer-ball-like shape and truncated icosahedron with 12 pentagons with C5–C5 single bonds and 20 hexagons with C5–C6 double bonds [75]. The average diameter of fullerene derivatives is 8.5 nm wide and C60 is currently the smallest and most commonly studied fullerene derivative [76]. After light activation, fullerene derivatives can generate ROS and are considered as potential therapeutic agents for PDT [77]. They contain an extended π-conjugated system, which offers the ability to absorb visible light. After light absorption, they form singlet excited states that undergo intersystem crossing to the triplet state. In this form, they are readily quenched by molecular oxygen and generate singlet oxygen, while others form free radicals [78]. Fullerene derivatives require less photobleaching, are more photostable, and are more ROS-inducing through the type I pathway than the commonly used tetrapyrrolic PS [79]. The fullerene derivatives are more effective in hypoxic tumors, as they induce more cytotoxic effects than singlet oxygens, which are produced by most PSs through a type II pathway [80]. Carbon-based materials are insoluble in water but the stability of their dispersions in an aqueous environment may be increased by incorporating them into water-soluble structures, such as liposomes, micelles, dendrimers, cyclodextrins, and nanoemulsion systems [79]. This approach protects their hydrophobic cores from the potential modifying effects of solvating agents and allows for limited contact with oxygen, which might cause a decreased PDT efficacy [81]. The novel design and characteristics (photosensitivity) allow C60 to also be utilized as a drug-carrier to improve the delivery to targeted areas. Meanwhile, C60 can actively contribute to the synergic therapeutic effect of PDT, chemotherapy, PTT, and others [82]. The general properties, advantages, and drawbacks of carbon nanomaterials are presented in Table 1.

The use of two-dimensional (2D) nanomaterials (NMs) as theranostic (therapeutic and diagnostic) agents is becoming popular as it improves the PDT outcomes for better cancer therapy [86]. One of the most studied 2D NMs is graphene and its derivatives or graphene-based materials (GBM), including few-layer graphene (FLG), graphene oxide (GO), reducing graphene oxide (rGO), nanographene oxide (NGO), and graphene quantum dots (GQDs) [87,88]. Graphene is made up of a single layer of carbon atoms forming a honeycomb-like structure, which has a high surface area of 2.630 m^2^/g, optimal thermal conductivity of approximately 5000 W/mK, approximate optical transparency of 2.3% of visible light, and a good room temperature quantum effect for electrons and holes [89]. GBMs offer an excellent carrier capacity and mobility due to the delocalization of its 2D plane sp^2^ hybridization that renders them potential candidates for improved delivery and theranostic applications [90]. In PDT, GBMs are mostly exploited due to their optimal loading efficiency and ability to absorb light in the near-infrared region of the visible spectrum; thus they are considered as promising anticancer agents, both in vivo and in vitro [91]. Besides the tumor-targeted drug delivery and high loading drug efficiency, they are considered as suitable materials for gene therapy using materials such as DNA, microRNA, short interfering RNA (siRNA), and anticancer agents [92]. With GBM-mediated PDT, cancer theranostics led to cancer-killing with limited effects on healthy tissues, where GBMs are considered as great therapeutic tools. Their supramolecular π–π stacking is extensively used in combination therapeutic approaches, as they are loading platforms for other agents, and thus the resulting systems enhance the therapeutic efficiency and synergetic killing ability [93,94].

Carbon nanotubes (CNTs) are closely related to GBMs as they are considered to be graphene- sheets rolled up into 1D hollow cylinders ranging between 1 to 100 nm wide. Many types of CNTs can be distinguished, where among them is the single-walled CNT (SWCNT) category and the two or more layers called multi-walled CNT (MWCNT) category. Due to the large π–π stacking systems present at the surfaces, CNTs may easily across various physiological barriers within the body to induce various responses, such as immunogenic and cytotoxic effects. As a result, they appear to have good phototherapeutic and anticancer activities [94,95]. Like graphene, CNTs have attracted attention and are widely studied due to their unique characteristics [73]. Similarly, they have strong light absorption in the NIR region, deep tissue penetration, and photothermal abilities. PTT acts via hyperthermia, which had been used for treating certain conditions by inducing selective cell death responses [96]. Therefore, CNTs can be utilized for PTT to damage cancer cells and reduce heat tolerance compared to normal cells. Cancer cells are highly proliferating and more prone to die from a shortage of supply of blood in the tumor [96]. In physiological conditions, CNTs are insoluble in water but oxidizing them with a strong acid generates carboxylic acid groups and CNT-derivatives that increase the stability of their dispersions in an aqueous medium. Among other advantages, CNTs are easily conjugated to other agents (e.g., hydrophobic drugs) in combination therapy to yield both PTT and PDT effects. Combining PTT with PDT or any other anticancer treatment could enhance the PTT-mediated anticancer effects with limited side effects to normal tissues through a controlled harsh thermal phenomenon, thus preventing inflammatory reactions and cancer metastasis. Using CNTs in combination therapy approaches is seen as a promising treatment that could induce better drug delivery and high therapeutic outcomes. Despite the therapeutic use and biomedical applications of CNMs, little is known about their induced-effects on different biological systems and cellular compartments.

So far, no clearance regarding the neurotoxicity, hepatotoxicity, nephrotoxicity, immunotoxicity, cardiotoxicity, genotoxicity, epigenetic toxicity, dermal toxicity, and carcinogenicity of CNMs had been reported [97]. However, several studies had shown that there is a certain level of toxicity associated with the use of 0D, 1D, and 2D CNMs in both normal and cancerous cells [98]. Like any effective therapeutic agent, CNMs have demonstrated capabilities in inducing cyto-damage responses, such as oxidative damage, immuno-dependent reactions, and cell death induction, all of which lead to cellular and nuclear fragmentation and destruction [99]. The biocompatibility and toxicity issues raised by CNMs represent important impediments for further uses, despite their proven capabilities in various biomedical applications [100]. As it stands, much more research is needed in order to determine the therapeutic indices of CNMs, as well as their safety protocols on biological systems and cellular compartments.

## 5. An Update on Chlorin-Based Photosensitizers Immobilized on Carbon Materials for Photodynamic and Photothermal Therapy

### 5.1. Carbon Dots

In order to describe the efficiency of chlorin-based photosensitizers immobilized on carbon dots for photodynamic and photothermal therapy against cancer, different parameters have been reported (2010–2019), such as the description of carbon material, concentration, light dose, incubation time, and type of cancer cell lines, where the results obtained are presented in Table 2.

In 2012, Huang and co-authors [101] reported the synthesis of multifunctional chlorin-e6-conjugated C-dots and their evaluation against cellosaurus (MGC 803) cells (Table 2, entry 1). In this protocol, the authors used the following parameters: a range of concentrations (0–50 μM), irradiation of 30 mW/cm^2^ for 3 min, and 24 h of incubation, obtaining a cell viability of 10%.

**Figure 9:**
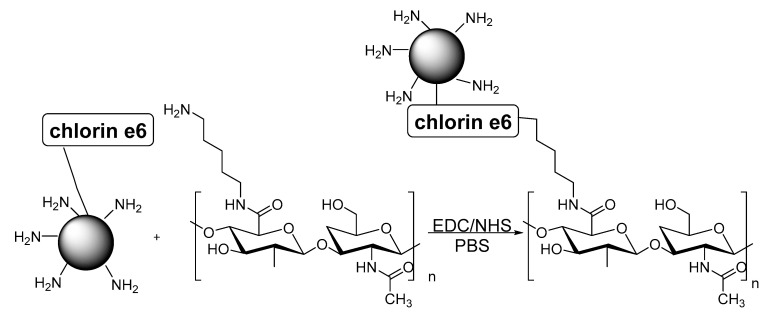
Bioconjugation of a chlorin-e6-functionalized carbon dot and diaminohexane-modified hyaluronate via the 1-ethyl-3-(3-dimethylaminopropyl)carbodiimide-*N*-hydroxysulfosuccinimide sodium (EDC-NHS) methodology.

In 2015, Beack and co-authors (Table 2, entry 2) reported the synthesis of a transdermal bioconjugate constituted of a carbon dot, chlorin e6 photosensitizer, and hyaluronate, and its application in a photodynamic protocol against melanoma skin cancer [102]. The authors prepared this bioconjugate using amide bond formation between a chlorin-e6-functionalized carbon dot and diaminohexane-modified hyaluronate in PBS as a solvent by using the 1-ethyl-3-(3-dimethylaminopropyl)carbodiimide-*N*-hydroxysulfosuccinimide sodium (EDC-NHS) methodology (Figure 9). The conjugation was confirmed using infrared spectroscopy [102].

For the in vitro photocytotoxicity assessment, a concentration of 1 µM, incubation of 4 h, and irradiation of 100 mW/cm^2^ (at 660 nm) for 10 min were used in B16F10 melanoma cells. The bioconjugate showed better results compared to chlorin e6 and chlorin-e6-functionalized carbon dots, which may have been due to its more effective uptake. For in vivo studies, the authors described that when this bioconjugate (carbon dot–chlorin e6 photosensitizer) was applied, the tumor volume increased without the laser irradiation but was suppressed under laser irradiation at 660 nm due to the photodynamic effect. In sum, this approach using transdermal delivery is safer and more effective than systemic delivery because it is locally accumulated in melanoma skin cancer [102].

In 2016, Wang and co-authors (Table 2, entry 3) reported the covalent linkage of chlorin e6 on polyethyleneimine-coated carbon nanodots by using the EDC strategy and its application in an in vitro photodynamic protocol against HeLa cancer cells [103]. In this study, the authors used the following parameters: chlorin e6 functionalized on polyethyleneimine-coated carbon nanodots (CDot-PEI-Ce6) (the final concentration of chlorin e6 was 2.6 μg/mL), under red light irradiation (15.5 mW/cm^2^), for 60 min, where a loss of cell viability (over 80%) was observed. This CDot-PEI-Ce6 presented high water solubility, biocompatibility, and high cellular uptake, as determined using flow cytometry and confocal laser scanning [103].

In order to develop a fluorescence imaging material that also shows photodynamic therapy performance against cancer, Hu and co-authors reported the preparation of layered double hydroxide (LDH) ultrathin nanosheets as a delivery vehicle to load chlorin e6 and carbon dots (Table 2, entry 4) [104]. The in vitro PDT studies using these chlorin e6 and carbon dots functionalized on layered double hydroxide ultrathin nanosheets (0–10 μg/mL) against HeLa cancer cells showed a good result (viability up to 9.6%) when using an incubation time of 24 h and a light fluence of 27 J/cm^2^ at 650 nm. Moreover, the authors found that this system (chlorin e6 + carbon dots + layered double hydroxides) presented satisfactory cellular imaging and a better photostability compared to chlorin e6 [104].

Aiming to reach the “ideal” balance between accumulation and clearance from the body, Liu and co-authors [105] reported the development of a new multifunctional nanomaterial: photosensitizer (chlorin e6)-loaded assembled carbon dots (Table 2, entry 5). Moreover, in this study, the authors used Gd^3+^ on the carbon dots’ surface for monitoring it via magnetic resonance and fluorescence imaging. For in vivo PDT studies, female BALB/c athymic nude mice at the ages of 6–8 weeks with weights of 18–22 g were used and the multifunctional nanomaterial synthesized was intravenously injected (3.0 mg/kg body weight (b.w.)) and irradiated at 633 nm using a power density of 0.5 W/cm^2^ (10 min) at 24 h after injection. On the 21st day after the PDT protocol, the tumor (formed by A549 cells that were transplanted subcutaneously) was analyzed and showed better performance than chlorin e6 in inhibiting tumor growth. Moreover, the authors observed that carbon dots underwent degradation into ultra-small particles under the acidic conditions in the tumor, and consequently, were easily cleared from the body [105].

### 5.2. Fullerene

In order to describe the efficiency of chlorin-based photosensitizers immobilized on fullerenes for photodynamic and photothermal therapy against cancer, different parameters have been reported (2010–2019), such as descriptions of the carbon material, concentration, light dose, incubation time, and type of cancer cell lines, where the results obtained are presented in Table 3.

**Figure 10:**
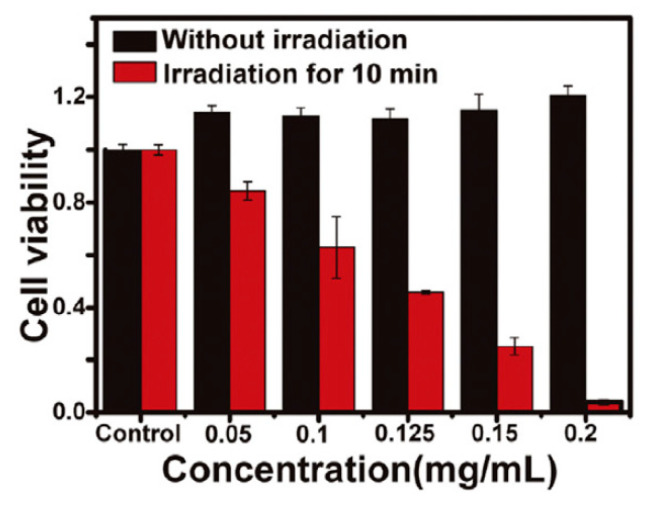
In vitro relative viabilities of A549 cells incubated with nanomaterial based on a tri-malonate derivative of fullerene (C70), with chlorin e6 as a photosensitizer and 1,10-diamino-4,7-dioxadecane (OEG2) as a linker at different concentrations, with 660 nm (20 mW/cm^2^ for 10 min) laser irradiation (red) or in the dark (black).

In 2016, Guan and co-authors [106] described the synthesis of a nanomaterial based on a tri-malonate derivative of fullerene (C70), with chlorin e6 as a photosensitizer and 1,10-diamino-4,7-dioxadecane (OEG2) as a linker (Table 3, entry 1). This nanocomposite based on a fullerene derivative was applied in an in vitro photodynamic procedure, where it was imaging-guided against human lung carcinoma/alveolar cell lines (A549 cells) using an incubation time of 3 h and irradiation (20 mW/cm^2^ for 10 min) at 660 nm. From the analysis of Figure 10, the authors obtained a cell viability of ~10% at 0.2 mg/mL of the nanocomposite (chlorin e6–functionalized fullerene C70).

Moreover, the authors evaluated this nanomaterial in an in vivo photodynamic study using female BALB/c mice (16–20 g) and a tumor model formed via subcutaneous injection of luciferase-expressing murine mammary carcinoma (4T1-luc) cells. For this in vivo protocol, the authors used a solution of 1 mg/mL of the developed material in saline as a solvent (200 μL, corresponding to a relative dose of 0.2 mg) with 4 h of incubation; then, the tumor region was irradiated for 10 min (at 660 nm, 100 mW/cm^2^) [106]. Overall, the authors described some advantages of the prepared nanocomposite: (i) high chlorin e6 loading efficiency (up to ≈57 wt%), (ii) good efficient absorption (red/NIR region), (iii) good cellular uptake for in vitro and in vivo studies, (iv) monitoring of tumor via imaging, (v) good biocompatibility, and (vi) full clearance from the body [106].

Recently, in 2020, Rybkin and co-authors reported a material obtained by covalently linking chlorin e6 to fullerene, which was characterized by means of absorption spectroscopy, steady-state/time-resolved fluorescence spectroscopy, dynamic light scattering, and an assessment of its photocytotoxicity (light fluence of 23 mW/cm^2^) against HeLa cancer cells (IC_50_ = 1.17 µM) (Table 3, entry 2) [107]. Moreover, the authors evaluated the influence of the linker and the presence of a Zn metal ion in the chlorin core on the physical/chemical properties and photodynamic efficiency; they observed that these functionalized carbon nanomaterials act via both type I and type II mechanisms. In this study, the authors also demonstrated that water-soluble fullerene derivatives, which were covalently linked to a hydrophobic dye, could be attractive for the creation of a highly active photosensitizer and as a platform for the synthesis of various nanoscale switch-off systems [107].

### 5.3. Carbon Nanotubes

In order to describe the efficiency of chlorin-based photosensitizers immobilized on carbon nanotubes for photodynamic and photothermal therapy against cancer, different parameters have been reported (2010–2019), such as descriptions of the carbon material, concentration, light dose, incubation time, and type of cancer cell lines, where the results obtained are presented in Table 4.

In 2011, Xiao and co-authors [108] reported the functionalization of single-wall carbon nanotubes with chlorin e6 and chitosan (Table 4, entry 1). This functionalized carbon nanomaterial showed a good efficiency (~10% cell viability at 30 µg/mL) against HeLa cells when a light dose of 20 J/cm^2^ and 24 h of incubation were used.

In 2016, in order to investigate the fundamental mechanisms of cell death and intracellular signaling cascades activated by PTT, PDT, and a combined therapy, Marangon and co-authors [109] prepared a dual system using multi-walled carbon nanotubes loaded via π–π stacking with the approved photosensitizer 5,10,15,20-tetrakis(3-hydroxyphenyl)chlorin (*m*THPC or Foscan^®^) (Table 4, entry 2). For the PDT and/or PTT procedures, the authors used the following parameters: *m*THPC-immobilized on carbon nanotubes at 0–20 µg/mL and at 650 nm (125 mW/cm^2^) for 300 s for PDT and/or at 808 nm (2.3 W/cm^2^) for 200 s for PTT. The authors found that the dual system prepared through multi-walled carbon nanotubes loaded via π–π stacking with the *m*THPC made the photosensitizer more photostable outside the cells, reduced non-specific diffusion of the non-functionalized photosensitizer, promoted cellular uptake, and promoted photothermal activation using the 808 nm irradiation [109]. This paper described and discussed the molecular mechanisms of PDT, PTT, and their combination by using flow cytometry, proteomics, and genomic analysis for the first time [109]. Overall, different mechanisms of cell death were evidenced depending on the photosentizer sub-localization and irradiation conditions (light dose), culminating synergistically to the apoptosis. Moreover, the authors studied the intracellular signaling cascades that were activated by PTT and PDT using cytometry, proteomics, and genomics. They demonstrated that single PDT, single PTT, and the combined treatment (PTT+PDT) elicited a programmed cell death that was instigated by oxidative stress [109].

In 2016, Xie and co-authors [110] described the combination of chlorin e6 with albumin, followed by its loading onto the surface of Evans blue-modified single-walled carbon nanotubes by exploiting the high affinity between albumin and Evans blue (Table 4, entry 3). For the in vitro PDT/PTT evaluation, cell viability of over 10% (mouse squamous carcinoma cell line SCC-7) was observed when PTT and PDT were combined when 630 nm (0.15 W/cm^2^ for 1 min) and 808 nm (1 W/cm^2^ for 2 min) was used. Moreover, for in vivo studies, at the same PDT/PDT parameters, tumors were virtually eradicated. Overall, the authors concluded that the use of combined PTT and PDT therapy can significantly improve treatment outcomes without recurrence, and Evans blue functionalization on carbon nanotubes can be easily extended to other nanomaterials [110].

In 2018, Yin and co-authors [111] developed a smart nanoplatform for cancer diagnosis and treatment via the functionalization of MnO_2_-coated carbon nanotubes with chlorin e6 (CMC) (Table 4, entry 4). The photothermal properties of the prepared nanoplatform were studied using a laser at 808 nm, where the temperature reached more than 50 °C, which is enough to combat cancer cells. For in vitro experiments, the efficiency of MnO_2_-coated carbon nanotubes with chlorin e6 was evaluated in HeLa cells using 24 h of incubation and irradiation at 633 nm, presenting an IC_50_ of 0.58 mg/mL. Furthermore, the in vivo synergistic cancer therapy was evaluated using nude mice (with xenograft HeLa cell line tumors), at various concentrations (0–1 µg/mL), irradiated with a laser for 10 min (1.0 W/cm^2^) at 24 h post-injection, and tumor volumes were determined every 2 days. For the CMC-treated group, the authors found that the tumors were eliminated (decrease of up to 100% of tumor volume). According to the authors, by combining photothermal and fluorescence imaging properties, the prepared nanoplatform presents great potential for dual imaging-guided synergistic PDT/PTT [111].

### 5.4. Graphene

In order to describe the efficiency of chlorin-based photosensitizers immobilized on graphene for photodynamic and photothermal therapy against cancer, different parameters have been reported (2010–2019), such as descriptions of the carbon material, concentration, light dose, incubation time, and type of cancer cell lines, where the results obtained are presented in Table 5.

In 2011, Tian and co-authors described the preparation of chlorin e6 loaded onto polyethylene glycol (PEG)-functionalized graphene oxide via supramolecular π–π stacking [93]. This nanomaterial was applied in a human nasopharyngeal epidermal carcinoma κB cell line by using the following parameters: concentration of photosensitizer (0.00138–0.011 mg/mL), 0.1 W/cm^2^ (10 min) of irradiation, and 24 h of incubation. The authors obtained a cell viability of 10% at 0.011 mg/mL (Table 5, entry 1).

Furthermore, also in 2011, Huang and co-authors [112] described the functionalization of folic-acid-conjugated graphene oxide with chlorin e6 (Table 5, entry 2). This nanomaterial was evaluated in cellosaurus (MGC803) cells using a range of concentrations (0–100 μM), irradiation of 30 mW/cm^2^ for 10 min, and 48 h of incubation, which resulted in a cell viability of ~10%.

Huang and co-authors [113] described the use of graphene oxide coated with polyvinylpyrrolidone and linked with a targeting peptide (Table 5, entry 3). Then, this nanomaterial was functionalized with chlorin e6 via the hydrophobic interactions of π–π stacking. The in vitro PDT evaluation of this nanomaterial (0–50 µM) was performed using human gastric mucinous adenocarcinoma (MGC803) cells, laser irradiation at 671 nm (30 mW/cm^2^) for 3 min, and an incubation time of 24 h. As a result, the authors observed complete cell killing after illumination when the synthesized nanosystem was used. Moreover, this nanosystem was able to increase the accumulation of chlorin e6 in tumors when compared to non-functionalized chlorin e6 [113].

In 2015, Liu and co-authors [114] described a simple sonication method (liquid-phase exfoliation) for the functionalization of graphene with chlorin e6 (Table 5, entry 4). For the in vitro PDT experiments, the authors used HeLa cells and evaluated the free chlorin e6 and chlorin-e6-functionalized graphene in different concentrations (0–0.20 µg/mL), with an incubation time of 24 h and irradiation at 660 nm (power density of 0.14 W/cm^2^) for 2 min. The authors found that free chlorin e6 exhibited negligible photocytotoxicity to HeLa cells when used in 0.20 µg/mL, while significant photocytotoxicity was observed in the presence of chlorin-e6-functionalized graphene at a Ce6 concentration of more than 0.050 µg/mL. In fact, this work described a simple method for graphene functionalization and functionalized graphene was presented as a promising nanomaterial for in vitro PDT against HeLa cells [114].

Furthermore, also in 2015, Zeng and co-authors [115] described the synthesis of polyethylene-glycol-functionalized graphene oxide followed by functionalization with branched polyethyleneimine (Table 5, entry 5). Then, this material was loaded with chlorin e6 via the hydrophobic interactions of π–π stacking. The authors found that this chlorin-e6-functionalized graphene showed a high PDT efficiency (up to 10% of viability at 2.0 µM) using HeLa cells, irradiation at 662 nm (0.2 W/cm^2^) for 5 min, and was also an excellent lysosome-targeting material [115].

In 2016, Zeng and co-authors [116] described the preparation of folic-acid-conjugated polyethylenimine-modified PEGylated nanographene for the targeted delivery of chlorin e6 (Table 5, entry 6). The authors evaluated this nanocomposite against (HeLa cells) using a concentration of 0.5–10 µg/mL, irradiation of 200 W/cm^2^ for 5 min, and 24 h of incubation. According to the authors, a cell viability of ~15% was obtained at 1.0 µM.

In 2016, Cao and co-authors [117] reported the synthesis of nanographene that was chlorin-e6-loaded and PEGylated, characterized using UV-vis, and applied in PDT, PTT, or a PDT/PTT combination (Table 5, entry 7). Moreover, a dual-modal MRI approach was applied for the monitoring and prognosis of phototherapies. For in vitro experiments, the authors evaluated three different groups against 4T1 cells: (i) PDT group—chlorin-e6-loaded onto PEGylated nanographene (0–2.0 µM), incubation time of 24 h, and irradiation at 660 nm (0.1 W/cm^2^ for 10 min); (ii) PTT group—chlorin-e6-loaded onto PEGylated graphene (0–2.0 µM), incubation time of 24 h, and irradiation at 808 nm (1 W/cm^2^ for 3 min); (iii) combined PDT and PTT group—chlorin-e6-loaded onto PEGylated graphene (0–2.0 µM), incubation time of 24 h, and irradiation at 660 and 808 nm (0.1 W/cm^2^ for 10 min) and (1 W/cm^2^ for 3 min). To evaluate this nanomaterial in the in vivo experiments, chlorin-e6-loaded and PEGylated nanographene (2 mg/mL per kg b.w.) was injected intravenously, followed by irradiation of the tumors (PTT group: at 808 nm, 1.5–2.5 W/cm^2^; PDT group: at 660 nm, 0.2 W/cm^2^; combination PDT + PTT: for 10 min at 808 nm followed by 10 min at 660 nm). Overall, for the in vitro and in vivo studies, the PDT/PTT group exhibited the highest cell death at all concentrations [117].

In 2017, Shim and co-authors [118] described the linking of 30-amino-acid claudin 4-binding peptide and *Clostridium perfringens* enterotoxin to chlorin e6 using poly(ethylene glycol) as a spacer and anchored to graphene oxide nanosheets (Table 5, entry 8). Regarding its application in PDT, the authors evaluated the effects on U87 cells, at a concentration of 50 µmol/L, an incubation time of 1 h, and using a laser at 808 nm (1.5 W). The authors observed a synergistic anticancer effect when the functionalized graphene oxide nanosheets were used to treat cells upon irradiation at two wavelengths (660 nm and 808 nm). Singlet oxygen production was observed to reach the highest value after 20 min of exposure at 660 nm (PDT procedure). The authors concluded that dual therapy (PTT and PDT) should be optimized to provide the highest therapeutic effect but it is a promising tool against different kinds of cell lines.

In 2018, Kim and collaborators described the synthesis of graphene oxide conjugated with chlorin e6 and methoxy poly(ethylene glycol) [119] (Table 5, entry 9). This nanomaterial was evaluated in a cellosaurus cell line using different concentrations (0–2.5 µg/mL), a light dose of 2.0 J/cm^2^, and 24 h of incubation, resulting in a cell viability of 10%. Moreover, the authors observed that the chlorin e6 release from graphene oxide was faster in the presence of glutathione, indicating that graphene oxide displays redox responsiveness.

Furthermore, also in 2018, Gulzar and co-authors [86] reported the development of core–shell-structured upconversion nanoparticles with graphene oxide, which was then loaded with chlorin e6; their evaluation in HeLa cells used concentrations of 25–800 µg/mL, irradiation of 0.72 W/cm^2^ (at 808 nm) for 10 min, and 24 h of incubation (Table 5, entry 10). The authors obtained a cell viability of ~15% at 800 µg/mL of the prepared nanomaterial.

In 2019, Kang and co-authors [120] promoted the functionalization of graphene oxide with a derivative purpurin-18-N-ethylamine chlorin e6 via non-covalent and covalent methodologies (Table 5, entry 11). The authors evaluated the PDT efficiency of functionalized graphene oxide (non-covalent and covalent) by using the following parameters: irradiation of 2 W/cm^2^ for 15 min and an incubation time of 3–24 h against human lung adenocarcinoma (A549) cells. The functionalized graphene oxide (non-covalent and covalent) showed IC_50_ values at 3, 12, and 24 h incubation of 0.69 and 0.22, 0.48 and 0.21, and 0.31 and 0.20 µM, respectively. Nevertheless, the non-covalently bound complex presented lower dark toxicity than the covalently bound complex [120]. According to the authors, the lower IC_50_ values of the non-covalently bound complex may have been because of its faster delivery effect based on the rapid intracellular release of the derivative purpurin-18-N-ethylamine chlorin e6 from the graphene oxide complex [120].

## 6. Conclusions and Future Perspectives

Over the years, conventional therapies have failed to effectively treat cancer; as a direct consequence, the condition has become one of the major causes of mortality worldwide. New technological approaches and strategic targeting to achieve better management of the condition, as well as early and proper diagnosis, therapy, and limited side-effects, are all required. Certain treatments are effective at killing cancer but also causing damage to healthy, neighboring tissues.

PDT procedures are able to combine a high efficacy against cancer cells without any serious damage to patients. Furthermore, its handling should be as easy as possible, using a low molar concentration of photosensitizer, and a low light dose. In this review paper, we emphasized the use of chlorin photosensitizers, which is a type of bioinspired tetrapyrrolic macrocycle. In the 1980s, chlorins were introduced as photosensitizers in PDT since they showed good accumulation in tumors, absorption at longer wavelengths (NIR), and fast clearance from the body. Nowadays, a family of synthetic and natural chlorin photosensitizers has been applied in PDT against cancer and other diseases.

In this regard, the combination of chlorin-based photosensitizers (especially chlorin e6) with carbon nanomaterials (carbon dots, carbon nanotubes, fullerene, and graphene) has been demonstrated as an effective method for diagnosis, as nano delivery systems, and against tumors due to PDT and PTT actions. Moreover, the use of a photothermal approach can overcome the obstacle of oxygen dependence that accompanies by PDT. We can emphasize that the effect of PDT and PTT are dependent on the type of carbon material, different preparation methods, morphologies, and modification methods.

Besides the high efficiency of chlorin-functionalized carbon nanomaterials in PDT/PTT applications, some challenges need to be overcoming with future studies: (i) the toxicity of carbon nanomaterials needs to be systematically evaluated concerning their size, functionalization, purity, and purification methods; (ii) the biodistribution, pharmacokinetics, and clearance from the body need to be studied and compared using different carbon nanomaterials and chlorins; (iii) the influence of the functionalization of carbon nanomaterials surfaces on PDT/PTT efficiency and toxicity needs to be evaluated and compared; (iv) the development of simple synthetic methods for the functionalization of carbon nanomaterials with photosensitizers (combined therapy) is required. In this review paper, we have demonstrated that the application of combined therapy (photothermal and photodynamic) produces a synergic effect for combating cancer cells (in vitro and in vivo studies).

## Figures and Tables

**Table 1:** General properties, advantages, and drawbacks of some carbon nanomaterials [83,84,85].

Entry	Carbon Nanomaterial	Properties	Advantages	Drawbacks
1	Carbon dot	D ^1^ = 0, strong optical absorption in the UV region (260–320 nm)	Low toxicity, excellent photoluminescence, good hydrophilicity, small size (below 10 nm), easy synthesis, good electrochemiluminescence, high stability in physiological media, good fluorescent property, biocompatible	Low solubility in physiological media, aggregation
2	Fullerene	D = 0, H ^2^ = mostly sp^2^, E.S.A. ^3^ = 80–90, T.C. ^4^ = 0.4, E.C. ^5^ = 10^−10^, T ^6^ = elastic, hardness = hard	Low toxicity, biocompatible	Low solubility in physiological media, aggregation
3	Carbon nanotube	D = 1, H = mostly sp^2^, E.S.A. = ~1300, T.C. = 3500, E.C. = structure-dependent, T = flexible, elastic, hardness = hard	Low toxicity, high conductivity, high chemical stability and sensitivity, high electron-transfer rate, biocompatible, strong NIR light absorption	Low solubility in physiological media, aggregation, low homogeneous size
4	Graphene	D = 2, H = sp^2^, E.S.A. = ~1500, T.C. = 4850–5300, E.C. = ~2000, T = flexible, elastic, hardness = uppermost	Low toxicity, high sensitivity, large surface area, inherent size- and shape-dependent optical properties, unique physicochemical behavior, biocompatible	Low solubility in physiological media, aggregation

^1^ D = dimensions, ^2^ H = hybridization, ^3^ E.S.A. = experimental specific surface area (m^2^ g^−1^); ^4^ T.C. = thermal conductivity (W m^−1^ K^−1^); ^5^ E.C. = electrical conductivity (S cm^−1^); ^6^ T = tenacity.

**Table 2:** Parameters used for chlorin-based photosensitizers immobilized on carbon dots as photosensitizers against cancer.

Entry	Carbon Material	Concentration	Irradiation	Incubation Time	Cancer Cell Lines	Results	Ref
1	Chlorin e6–conjugated C-dots	0–50 µM	30 mW/cm^2^ (3 min)	24 h	MGC803 cells	~10% (cell viability)	[101]
2	Chlorin e6–carbon dot	1 µM	100 mW/cm^2^ (10 min)	4 h	B16F10 cells	~10% (cell viability)	[102]
3	Chlorin e6–polyethyleneimine-coated carbon nanodots	2.6 μg/mL	15.5 mW/cm^2^ (60 min)	24 h	HeLa cancer cells	20% (cell viability)	[103]
4	Layered double hydroxides–chlorin e6–carbon dots	0–10 μg/mL	27 J/cm^2^	24 h	HeLa cancer cells	9.8% (cell viability)	[104]
5	Chlorin e6–carbon dot	3.0 mg/kg body weight (b.w.)	0.5 W/cm^2^ (10 min)	24 h	BALB/c athymic nude mice (A549 cells)	Volume tumor was decreased (up to 80%)	[105]

**Table 3:** Parameters used for chlorin-based photosensitizers immobilized on fullerenes as a photosensitizer against cancer.

Entry	Carbon Material	Concentration	Irradiation	Incubation Time	Cancer Cell Lines	Results	Ref
1	Fullerene (C70)–chlorin e6	0.05–0.2 mg/mL	20 mW/cm^2^ at 660 nm (10 min)	3 h	A549 cells	~10% (cell viability)	[106]
2	Fullerene–chlorin e6	10 mM	23 mW/cm^2^ at 630 nm (30 min)	24 h	HeLa cancer cells	IC_50_ = 1.17 µM	[107]

**Table 4:** Parameters used for chlorin-based photosensitizers immobilized on carbon nanotubes as a photosensitizer against cancer.

Entry	Carbon Material	Concentration	Irradiation	Incubation Time	Cancer Cell Lines	Results	Ref
1	Single-wall carbon nanotubes–chlorin e6–chitosan	5–100 µg/mL	20 J/cm^2^	24 h	HeLa cells	~10% cell viability at 30 µg/mL	[108]
2	Multi-walled carbon nanotubes–*m*THPC	8–20 µg/mL	125 mW/cm^2^ at 650 nm (300 s) or 2.3W/cm^2^ at 808 nm (200 s)	3 h	Human ovarian carcinoma SKOV-3 cells	~10% (cell viability)	[109]
3	Chlorin e6 with albumin–single-walled carbon nanotube	1–50 mg/mL	0.15 W/cm^2^ at 630 nm (1 min) and 1 W/cm^2^ at 808 nm (2 min)	12 h	Mouse squamous carcinoma cell line SCC-7	10% cell viability	[110]
4	MnO_2_-coated carbon nanotubes with chlorin e6	0.5–1 µg/mL	1.0 W/cm^2^ at 660 nm (5 min)	24 h	HeLa cells	IC_50_ of 0.58 mg/mL	[111]

**Table 5:** Parameters used for chlorin-based photosensitizers immobilized on graphene as a photosensitizer against cancer.

Entry	Carbon Material	Concentration	Irradiation	Incubation Time	Cancer Cell Lines	Results	Ref
1	Graphene oxide–polyethylene glycol–chlorin e6	0.00138–0.011 mg/mL	0.1 W/cm^2^ (10 min)	24 h	Human nasopharyngeal epidermal carcinoma KB cell line	~10% cell viability	[93]
2	Folic-acid-conjugated graphene oxide–chlorin e6	0–100 μM	~30 mW/cm^2^ (10 min)	48 h	MGC803 cells	~10% cell viability	[112]
3	Graphene oxide– polyvinylpyrrolidone–chlorin e6	0–50 µM	30 mW/cm^2^ (3 min)	24 h	MGC803 cells	complete cell killing	[113]
4	Graphene–chlorin e6	0–0.20 µg/mL	0.14 W/cm^2^ (2 min)	24 h	HeLa cells	Up to 100% cell killing	[114]
5	Graphene oxide– polyethylene glycol–chlorin e6	0.25–2 µM	0.2 W/cm^2^ (5 min)	24 h	HeLa cells	10% cell viability	[115]
6	Folic-acid-conjugated polyethylenimine–PEGylated graphene–chlorin e6	0.5–10 µg/mL	200 W/cm^2^ (5 min)	24 h	HeLa cells	~15% cell viability	[116]
7	PEGylated nanographene–chlorin e6	0–2.0 µM	0.1 W/cm^2^ (10 min)	24 h	4T1 cells	Complete cell killing	[117]
8	Graphene oxide– polyethylene glycol–chlorin e6	50 µmol/L	1.5 W at 808 nm	1 h	U87 cells	10% cell viability	[118]
9	Chlorin e6–PEG-conjugated graphene oxide	0–2.5 µg/mL	2.0 J/cm^2^	24 h	CCA cells	10% cell viability	[119]
10	Up-conversion nanoparticles–graphene oxide–chlorin e6	25–800 µg/mL	0.72 W/cm^2^ (10 min)	24 h	HeLa cells	15% cell viability	[86]
11	Graphene oxide–chlorin e6	1.0 µM	2 W/cm^2^ (15 min)	3–24 h	A549 cells	IC_50_ = 0.69 at 3 h	[120]
